# Supplementary material for: Role and mechanism of NCAPD3 in promoting malignant behaviors in gastric cancer
Source: Front Pharmacol. 2024 Apr 22;15:1341039. doi: 10.3389/fphar.2024.1341039 (PMC11070777; doi:10.3389/fphar.2024.1341039)
Supplement: Supplementary file 11 [file DataSheet2.ZIP › GSEA/immunologic/my_analysis.Gsea.1599462003817/GSE360_L_DONOVANI_VS_B_MALAYI_HIGH_DOSE_DC_UP.html]

Details for gene set GSE360\_L\_DONOVANI\_VS\_B\_MALAYI\_HIGH\_DOSE\_DC\_UP[GSEA]

|  || Dataset | filtered\_dataset.sample\_info.cls#WT\_versus\_NCAPD3\_MUT |
| Phenotype | sample\_info.cls#WT\_versus\_NCAPD3\_MUT |
| Upregulated in class | NCAPD3\_MUT |
| GeneSet | GSE360\_L\_DONOVANI\_VS\_B\_MALAYI\_HIGH\_DOSE\_DC\_UP |
| Enrichment Score (ES) | -0.5044843 |
| Normalized Enrichment Score (NES) | -2.5200007 |
| Nominal p-value | 0.0 |
| FDR q-value | 0.013518827 |
| FWER p-Value | 0.096 |
Table: GSEA Results Summary

  

Fig 1: Enrichment plot: GSE360\_L\_DONOVANI\_VS\_B\_MALAYI\_HIGH\_DOSE\_DC\_UP      
 Profile of the Running ES Score & Positions of GeneSet Members on the Rank Ordered List

  

| SYMBOL | TITLE | RANK IN GENE LIST | RANK METRIC SCORE | RUNNING ES | CORE ENRICHMENT || 1 | 637 | BID | 478 | 0.487 | -0.3033 | No |
| 2 | 219 | ALDH1B1 | 638 | 0.405 | -0.3835 | No |
| 3 | 2114 | ETS2 | 703 | 0.375 | -0.3978 | No |
| 4 | 7357 | UGCG | 852 | -0.254 | -0.4829 | Yes |
| 5 | 4502 | MT2A | 863 | -0.277 | -0.4666 | Yes |
| 6 | 8837 | CFLAR | 865 | -0.278 | -0.4437 | Yes |
| 7 | 6515 | SLC2A3 | 873 | -0.283 | -0.4247 | Yes |
| 8 | 3005 | H1F0 | 880 | -0.294 | -0.4040 | Yes |
| 9 | 694 | BTG1 | 901 | -0.326 | -0.3908 | Yes |
| 10 | 4616 | GADD45B | 904 | -0.330 | -0.3641 | Yes |
| 11 | 6273 | S100A2 | 942 | -0.365 | -0.3598 | Yes |
| 12 | 4973 | OLR1 | 944 | -0.366 | -0.3295 | Yes |
| 13 | 3665 | IRF7 | 956 | -0.375 | -0.3056 | Yes |
| 14 | 2146 | EZH2 | 1001 | -0.406 | -0.3028 | Yes |
| 15 | 54541 | DDIT4 | 1133 | -0.502 | -0.3546 | Yes |
| 16 | 1263 | PLK3 | 1134 | -0.502 | -0.3120 | Yes |
| 17 | 7444 | VRK2 | 1145 | -0.509 | -0.2760 | Yes |
| 18 | 27074 | LAMP3 | 1158 | -0.522 | -0.2403 | Yes |
| 19 | 7128 | TNFAIP3 | 1198 | -0.573 | -0.2197 | Yes |
| 20 | 3892 | KRT86 | 1313 | -0.724 | -0.2404 | Yes |
| 21 | 1839 | HBEGF | 1328 | -0.751 | -0.1867 | Yes |
| 22 | 1116 | CHI3L1 | 1339 | -0.777 | -0.1280 | Yes |
| 23 | 7422 | VEGFA | 1346 | -0.788 | -0.0654 | Yes |
| 24 | 4312 | MMP1 | 1407 | -1.304 | 0.0022 | Yes |
Table: GSEA details [plain text format]

  

Fig 2: GSE360\_L\_DONOVANI\_VS\_B\_MALAYI\_HIGH\_DOSE\_DC\_UP      
 Blue-Pink O' Gram in the Space of the Analyzed GeneSet

  

Fig 3: GSE360\_L\_DONOVANI\_VS\_B\_MALAYI\_HIGH\_DOSE\_DC\_UP: Random ES distribution      
 Gene set null distribution of ES for **GSE360\_L\_DONOVANI\_VS\_B\_MALAYI\_HIGH\_DOSE\_DC\_UP**
